# Editorial: Nutrition at the Crossroads: Food at the Intersection of Environmental, Economic, and Social Sustainability

**DOI:** 10.3389/fnut.2019.00158

**Published:** 2019-10-01

**Authors:** Kurt A. Rosentrater, Laetitia Palmade, Elif Kongar

**Affiliations:** ^1^Departments of Agricultural and Biosystems Engineering, and Food Science and Human Nutrition, Iowa State University, Ames, IA, United States; ^2^Département Génie Biologique et Agroalimentaire, Polytech Montpellier, Université de Montpellier, Montpellier, France; ^3^Departments of Technology Management and Mechanical Engineering, University of Bridgeport, Bridgeport, CT, United States

**Keywords:** food, sustainability, environment, water, energy

Societies around the world are at a critical juncture. As the planetary population grows, there are increasing demands for expanding available food, more nutritious and healthy foods, and contemporaneously the need for greater efficiencies and decreased impacts upon the environment.

The sustainability of food production systems is a complex issue that requires a global and multidisciplinary approach, combining not only agronomy, ecology, nutrition, epidemiology, processing, energy use, but also marketing and sociology. It is within this context that this special issue aims to illustrate, through review articles, case studies, as well as modeling and simulation studies, some means of understanding the integration of food, in a broad sense, within our environment and society.

For example, currently consumption of meat products is being challenged both environmentally and nutritionally, and the transition to alternative protein sources is being encouraged. However, a better understanding of the links between livestock breeding, feeding, environmental impacts, and the nutritional quality of meat and dairy products produced could improve the sustainability of these food choices. On the other hand, consumer demand for a healthier and more sustainable diet, which may lead to the consumption of new products, such as insects, requires the implementation of specific dissemination strategies and government programs, integrating marketing, economic, and psychological aspects. Technological challenges are also addressed in this special issue, with an overview of various strategies adopted by many food companies to reduce energy and water consumption. Among them, the use of new non-thermal technologies is a promising way to improve the sustainability of food processing by reducing environmental impacts. In addition, key issues concerning the development of packaging with reduced ecological footprints are also presented. A focus is also placed on the importance for companies to integrate environmental and social sustainability markers into industrial performance indicators.

Although not exhaustive, we have attempted to cover multiple facets of food systems in this Research Topic. For example, several articles focus on food processing factories and operations; others focus on ingredients and food choices; while others discuss business operations. Some have as a primary focus empirical data, others utilize survey information, while others pursue simulation and modeling. Nikmaram and Rosentrater provide an overview of recent technologies and advances in energy and water efficiencies in food processing factories. Picart-Palmade et al. provide a deep discussion about various non-thermal technologies, and how these are improving the efficiencies of food processing operations. Guillard et al. discuss advances in packaging materials and how these are improving the sustainability of food systems, especially within the context of circular economies. Dohmen and Raman focus on human diets, education, prices, and food choices. Provenza et al. explore which type of beef and dairy production are more sustainable—pasture or feedlot-raised—as method of raising/feeding will impact environmental impacts. Berger et al. investigate insects as a source of proteins, and whether they can live up to their promise as a sustainable food source for humans. Finally, Duman et al. model the integration of environmental and social metrics using a Balanced Scorecard approach for food production business operations.

This issue is especially timely since, in an era of big data and predictive analysis, managerial reliance on modeling and simulation have never been greater. Today, the wide availability of data collection, storage, and dissemination tools have fostered efforts to create more intelligent, better informed, and data-driven decision making platforms. Collectively these can provide a means for internal and external optimization of business operations, and can ultimately improve the environmental and societal impacts of products and processes. Simulation models and analysis tools also allow tracking and recording of real-time data that can be inputted into performance evaluation systems. In integrated systems these types of models and analyses not only provide a better understanding of the issues, but also make it possible for internal and external criteria, and their interrelations, to be assessed in the analysis. Thus, resource utilization, risk identification, forecasting, and performance evaluation can be dynamically quantified.

Indeed, assessing and improving the global food system is highly complex, even when considered from a regional viewpoint or an industry-specific standpoint. This Research Topic covers some facets of the food industry, but not all. For the interested reader, many other references are also available. A few of these include Burlingame and Dernini (1), SUSFANS (2), Tuomisto (3), and Willett et al. (4).

Additional resources can be found in other Research Topics that have been published in Frontiers in Sustainable Food Systems, including livestock nutrition, soil carbon, modeling agro-ecosystems, urban agriculture, food waste, and many more. These can be found at https://www.frontiersin.org/journals/sustainable-food-systems#research-topics.

We would like to thank all of the contributing authors to this Research Topic. It is encouraging to see such innovative work. We hope that you, the reader, also find this Research Topic interesting and useful. And hopefully this helps to spur the conversation about food, systems, and steps that can be taken to improve environmental and social sustainability as the industry tries to achieve the goals of an abundant, nutritious, safe, and environmentally-friendly food supply.

## Author Contributions

All authors listed have made a substantial, direct and intellectual contribution to the work, and approved it for publication.

### Conflict of Interest

The authors declare that the research was conducted in the absence of any commercial or financial relationships that could be construed as a potential conflict of interest.
